# Perceptions About Augmented Reality in Remote Medical Care: Interview Study of Emergency Telemedicine Providers

**DOI:** 10.2196/45211

**Published:** 2023-03-28

**Authors:** Alana Dinh, Emily Tseng, Andrew Lukas Yin, Deborah Estrin, Peter Greenwald, Alexander Fortenko

**Affiliations:** 1 Medical College Weill Cornell Medicine New York, NY United States; 2 Department of Information Science Cornell Tech New York, NY United States; 3 Department of Medicine Weill Cornell Medicine New York, NY United States; 4 Department of Computer Science Cornell Tech New York, NY United States; 5 Emergency Medicine NewYork-Presbyterian Hospital New York, NY United States

**Keywords:** augmented reality, telemedicine, telehealth, emergency medicine, education, mobile phone

## Abstract

**Background:**

Augmented reality (AR) and virtual reality (VR) have increasingly appeared in the medical literature in the past decade, with AR recently being studied for its potential role in remote health care delivery and communication. Recent literature describes AR’s implementation in real-time telemedicine contexts across multiple specialties and settings, with remote emergency services in particular using AR to enhance disaster support and simulation education. Despite the introduction of AR in the medical literature and its potential to shape the future of remote medical services, studies have yet to investigate the perspectives of telemedicine providers regarding this novel technology.

**Objective:**

This study aimed to understand the applications and challenges of AR in telemedicine anticipated by emergency medicine providers with a range of experiences in using telemedicine and AR or VR technology.

**Methods:**

Across 10 academic medical institutions, 21 emergency medicine providers with variable exposures to telemedicine and AR or VR technology were recruited for semistructured interviews via snowball sampling. The interview questions focused on various potential uses of AR, anticipated obstacles that prevent its implementation in the telemedicine area, and how providers and patients might respond to its introduction. We included video demonstrations of a prototype using AR during the interviews to elicit more informed and complete insights regarding AR’s potential in remote health care. Interviews were transcribed and analyzed via thematic coding.

**Results:**

Our study identified 2 major areas of use for AR in telemedicine. First, AR is perceived to facilitate information gathering by enhancing observational tasks such as visual examination and granting simultaneous access to data and remote experts. Second, AR is anticipated to supplement distance learning of both minor and major procedures and nonprocedural skills such as cue recognition and empathy for patients and trainees. AR may also supplement long-distance education programs and thereby support less specialized medical facilities. However, the addition of AR may exacerbate the preexisting financial, structural, and literacy barriers to telemedicine. Providers seek value demonstrated by extensive research on the clinical outcome, satisfaction, and financial benefits of AR. They also seek institutional support and early training before adopting novel tools such as AR. Although an overall mixed reception is anticipated, consumer adoption and awareness are key components in AR’s adoption.

**Conclusions:**

AR has the potential to enhance the ability to gather observational and medical information, which would serve a diverse set of applications in remote health care delivery and education. However, AR faces obstacles similar to those faced by the current telemedicine technology, such as lack of access, infrastructure, and familiarity. This paper discusses the potential areas of investigation that would inform future studies and approaches to implementing AR in telemedicine.

## Introduction

### Background

Telemedicine is defined as the use of communication and information technology to facilitate access to medical care or information [1]. *Telemedicine*, *telehealth*, and *virtual health* are terms commonly used interchangeably. *Telemedicine* focuses on clinical services, whereas *telehealth* entails remote health care services that can be clinical or nonclinical in nature, including public health and health care administration purposes [2]. *Virtual health* is an even broader term, encompassing any medical innovation or service that does not involve face-to-face interaction, including but not limited to remote visits, digital communication, real-time monitoring, and tools that increase patient access to care [3]. Although telemedicine has existed for decades, its use remained limited across physicians until the COVID-19 pandemic, during which changes in reimbursement and regulatory frameworks expedited the adoption of real-time audio-video platforms to provide medical services [4-6]. As the impact of telemedicine has reached a variety of specialties [7,8] and public awareness of its convenience has increased, many speculate that providers will continue to offer telemedicine options for some services in the future.

Telemedicine has been explored in a variety of uses, including but not limited to patient examination [7], teleconsultation [8], telesurgery [9] and medical education [10]. However, many users still do not view telemedicine as a substitute for in-person care; physicians cited limitations in diagnostic ability [11] and training [12-14], whereas patients cited limited interactivity and difficulty of access [15]. Advances in technology hope to address some of these barriers, with augmented reality (AR) showing considerable potential.

AR and virtual reality (VR) are 2 closely related yet distinct types of emerging technologies that fall under the umbrella of mixed reality technology. VR is defined as complete immersion, in which all the user experiences are synthetic, whereas AR is defined as a combination of the virtual world and real world [16]. Head-mounted devices (HMDs) using VR block the external world and replace it with a computer-generated environment, whereas devices implementing AR, which could be HMDs or software on a personal device, instead supplement the user’s perception of the real world by overlaying it with computer-generated audio, graphics, or video [17]. AR has been involved in 338 original studies related to medicine from 2012 to 2017 [18], with more recent studies demonstrating AR in real-time telemedicine contexts ranging from telerehabilitation [19] to inpatient consultation [20], telepathology [21], and remote plastic surgery education [22].

### Objective

As a specialty, emergency medicine has readily implemented technology to improve patient care and education, such as telemedicine-related solutions [2,23,24], point-of-care ultrasound [25], and simulation [26]. A 2019 review of AR technology in emergency medicine found 8 papers related to telemedicine including remote surgical treatments, triage, and telementoring [27], whereas more recent papers have similarly described the use of AR in remote communication for emergency responders and procedures [28,29]. In addition to support for disaster medicine [30], AR has also been used in other remote contexts such as teleultrasound [31] and the teaching of teamwork during high-risk scenarios [32]. Although these studies suggest that emergency medicine as a specialty is poised to further explore the intersection of AR and telemedicine, there is paucity of literature on the perspectives of emergency telemedicine providers regarding the subject.

In this study, we performed semistructured interviews to collect the perceptions and concerns of emergency telemedicine providers and identify potential applications for AR in telemedicine and anticipated barriers that may influence this unique integration. The results of this study may inform those in health care about AR’s potential role in real-time, remote medical communications and guide the future designs and priorities of innovators.

## Methods

### Research Questions

The research questions were as follows: (1) In what ways do emergency providers perceive AR being used in telemedicine? (2) What potentials and challenges do they anticipate from this intersection? and (3) How do they expect other providers and patients to react to the implementation of AR in telemedicine?

### Recruitment

This study sought to recruit emergency medicine providers with various experiences in using synchronous, audio-video telemedicine. Participants were expected to have at least 6 months of experience in using telemedicine in clinical practice or 1 month of experience in trialing a newly launched telemedicine platform supported by a medical center. The inclusion criteria were designed to represent both experienced telemedicine users and those who have adopted telemedicine briefly owing to institutional demand or the COVID-19 pandemic but may have discontinued its use. The use of AR technology was anticipated to be uncommon and therefore not a recruitment criterion. Instead, a snowball sampling technique was used to identify individuals with engagement or interest in AR technology.

In total, 8 qualifying individuals from a single large academic medical institution’s telemedicine center were invited to participate in the study. Overall, 75% (6/8) of those individuals responded and agreed to participate, and a snowball sampling method was used to identify any additional qualifying providers from the same or other academic medical institutions.

### Data Collection

Semistructured interviews were used to understand each participant’s unique experiences and perspectives regarding the intersection of telemedicine and AR technology. Owing to the COVID-19 pandemic and the variable locations of participants, interviews were conducted over a videoconferencing platform (Zoom; Zoom Video Communications) by a single interviewer (AD) to provide consistency, and the interviews lasted approximately 45 to 60 minutes each. The interviewer used a set of predetermined questions and follow-up probes (Multimedia Appendix 1) that could be divided into 3 sections. The first section focused on the participant’s past experiences, including medical background, telemedicine use, and history with AR and VR technology. Before discussing AR and VR exposure, participants were prompted to describe AR and VR in their own words and the distinction between the 2 terms was clarified as needed.

The second section began with video demonstrations of a prototype system for remote caregiving, with questions about the potential uses and drawbacks of the different AR features shown. Video demonstrations were used to exemplify for participants what the technology could do, given the relative novelty of AR in telemedicine. The prototype platform [33] was developed by an interdisciplinary team of technologists by using HMDs and depth sensors. In the videos, a local caregiver wears an HMD to visualize AR features used by a remote provider, including a 3D representation of the remote provider, gesture visualizations (3D pointing hands), annotations, and 2D images that were superimposed on the local environment. Meanwhile, the remote provider wears an HMD and holds 2 controllers to access the AR tools used to engage with the local caregiver. Through the HMD, the provider visualizes a live 3D representation of a local caregiver and patient. Although the provider’s reconstructed perspective leverages VR, the videos focused on the AR features as seen by the local caregiver, with AR and VR perspectives captioned in the videos for clarity. Although the prototype did not show the remote provider accessing the local caregiver’s perspective, the video demonstrations allowed interviewees to view the caregiver’s perspective directly. The ability to view the local perspective was also considered as a feature of AR and included for discussion during the interview.

**Figure 1:**
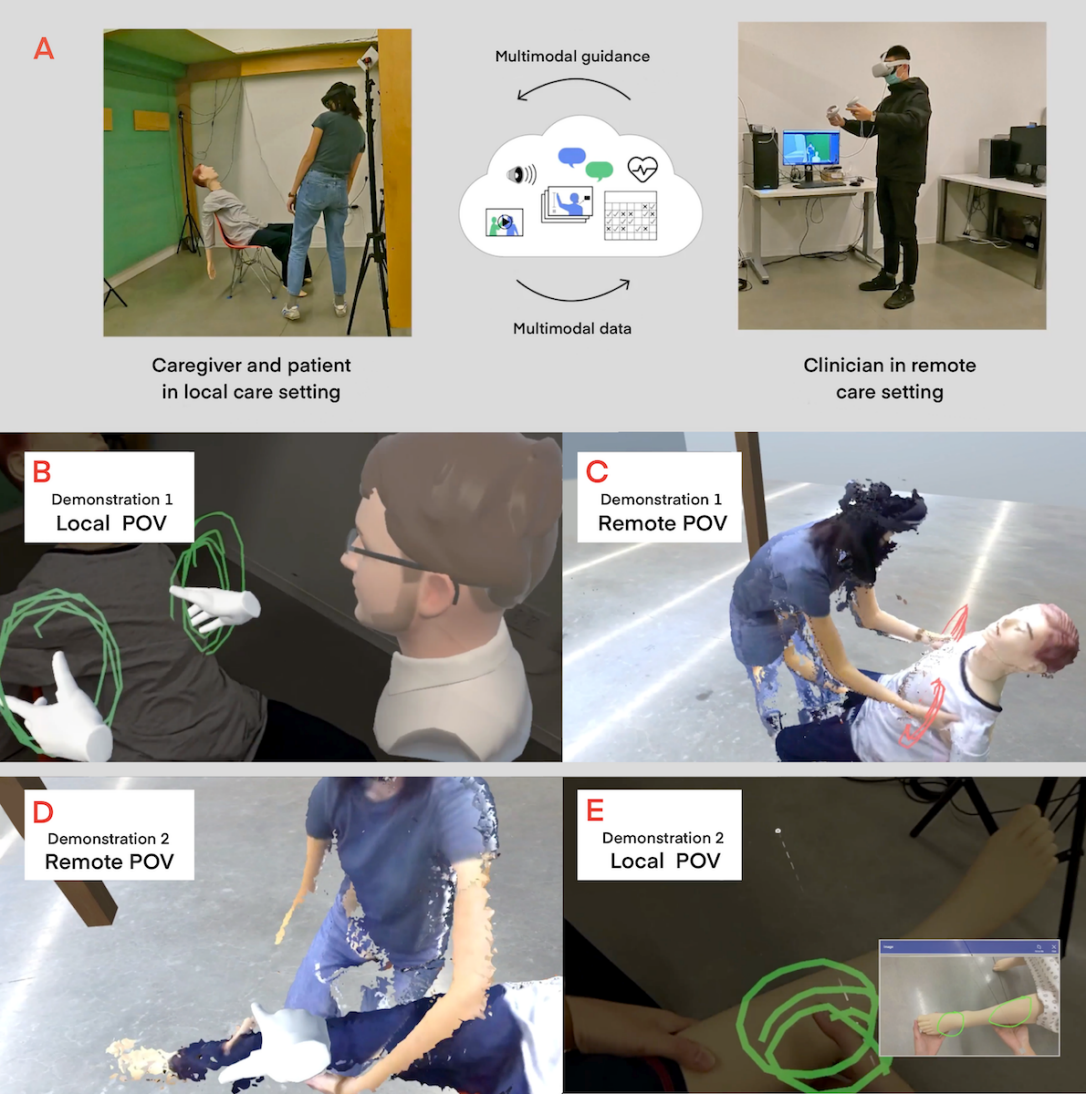
Prototype demonstration. (A) Information is shared between the caregiver with the patient (mannequin) at the local site and the provider at the remote site. (B) Circles and virtual representations of the provider overlay on the local perspective and show where the caregiver’s hands should be placed when lifting the patient. (C) The provider’s view of the caregiver as the lift is performed. (D) In the second demo, the provider’s virtual hand points to the patient’s leg while giving instructions to roll up the pants. (E) Overlaying the caregiver’s perspective are circles and a reference image to assist in performing a lower extremity edema assessment. POV: point of view.

In the first video, the remote provider uses annotations and gesture visualizations to demonstrate how to safely lift the patient from a sitting position to a standing position. In the second video, the remote provider uses annotations and a 2D image to demonstrate how to test for lower extremity edema (Multimedia Appendix 2). Figure 1 depicts the prototype and AR features demonstrated.

Although the interviewed participants were given the opportunity to reflect on their impressions from the videos, they were asked to provide their perspectives about the exemplified AR features with any setting and combination of users or equipment in mind. Given the current lack of standardization for AR devices, allowing participants to speak generally about AR technology in telemedicine would generate data that inform future designs and systems for remote health care delivery, rather than speaking about the specific setup and limitations of the prototype demonstrated. Participants were also prompted to describe additional features for AR that they would like to see.

Finally, the third section focused on the general usability, feasibility, and acceptability anticipated for AR in telemedicine for different contexts and users. Participants were instructed to answer the subsequent questions with any combination of users, equipment, features, and contexts in mind. All interviews were audio recorded. The audio recordings were then transcribed verbatim. Interview transcriptions generated in this study were deidentified before analysis, with personal information that could identify the participant removed where possible. Participants received no compensation for their participation.

### Data Analysis

Interview data were analyzed using a constructivist qualitative coding approach adapted from thematic coding [34]. Widely used in human-computer interaction [35], this approach allows a research team to analyze a data set by developing descriptive codes through multiple rounds of interpretation, which are then clustered into a set of themes that characterize the data. Each interview transcription was reviewed line by line and tagged with common concepts. Codes were developed to represent each concept, and an initial codebook was developed using codes from the first 6 transcriptions. New codes were added as subsequent transcriptions were analyzed, until thematic saturation, the point at which no new codes were generated, was reached. During the second stage of coding, initial codes were consolidated by 1 researcher (AD), and refined codes were categorized into subthemes and themes by 2 researchers (AD and ET), with disagreements resolved through discussion.

### Ethical Considerations and Consent to Participate

Participants provided informed consent to enroll in the study and for their interview to be audio recorded. This study received exempt status from the Cornell University institutional review board as part of an amendment under protocol number 1810008331.

## Results

### Participants

In total, 21 emergency medicine providers (n=11, 52% men and n=10, 48% women) affiliated with 10 academic medical institutions across Washington, District of Columbia, and 8 states (Indiana, New Hampshire, New York, Pennsylvania, Massachusetts, Maryland, Missouri, and South Carolina) participated in the study. The study population included 76% (16/21) adult emergency medicine attending physicians, 5% (1/21) physician assistant, and 19% (4/21) pediatric emergency medicine attending physicians. The average number of years of patient practice following completion of training (residency and fellowship) was 12.3 (SD 7.3; range 0-26) years. Of the 21 participants, 9 (43%) had pursued a fellowship in critical care, simulation, pediatric emergency medicine, or research.

The average number of years using synchronous, audio-video telemedicine in clinical practice was 3.9 (SD 2.4 years; range 1 month to 9 years) years. The most commonly reported platform used for telemedicine was Zoom (9/21, 43%), with 33% (7/21) of the participants having used >1 platform. Of the 21 participants, 5 (24%) participants reported trialing a newly launched telemedicine platform and stopping owing to the platform being discontinued, the platform no longer being required for practice, or moving away from the institution. When asked what percentage of time spent in patient care currently involves telemedicine (or during the period of active telemedicine use), participants reported an average of 27%, with 33% (7/21) of the individuals reporting an increase in percentage if administrative or research time was included, ranging from 33% to 100%. Overall, 38% (8/21) of the participants held leadership positions within their departments related to telehealth, including telemedicine, teleparamedicine, tele–intensive care unit, and digital health programs.

The time of first exposure to AR was generally reported in 2 patterns: 29% (6/21) of the participants reported hearing about AR around 2013 during the introduction of the Google Glass and 43% (9/21) reported the late 2010s as a result of educational conferences or apps related to gaming (eg, Pokemon Go). Of the 21 participants, 6 (29%) participants reported using AR or VR technology with a frequency of once every 3 months or more, whereas 4 (19%) other participants reported never having personally used AR or VR technology. Of the 21 participants, 11 (52%) participants reported using AR or VR in a health care context at least once, including conferences, research, and educational programs. A summary about the participants is shown in Table 1.

Thematic saturation was reached after the 16th transcript. The resulting codes (refer to the codebook in Multimedia Appendix 3) fell under 9 subthemes and 3 overarching themes discussed in the following section (Table 2).

**Table 1:** Participant demographics and experiences.

Participant	Occupation	Completed years of practice (after training)	Leadership roles in telehealth	Frequency of AR^a^ or VR^b^ use	Roles involving AR or VR
1	EM^c^ physician	7	N/A^d^	Weekly	Simulation center leadership
2	EM physician	23	Telehealth director	Monthly	N/A
3	EM physician	13	N/A	Minimal^e^	Simulation center faculty
4	EM physician	5	Teleparamedicine director	Minimal	N/A
5	EM physician	1	N/A	Quarterly	N/A
6	EM physician	7	N/A	Never	N/A
7	EM physician	21	N/A	Minimal	N/A
8	EM physician	13	Telehealth fellowship director	Minimal	Digital medicine educator
9	PEM^f^ physician	6	Pediatric telemedicine director	Minimal	N/A
10	EM physician	3	N/A	Minimal	N/A
11	EM physician assistant	15	N/A	Never	N/A
12	EM physician	16	Telehealth director	Minimal	N/A
13	EM physician	16	Virtual health director	Never	N/A
14	EM physician	15	N/A	Minimal	N/A
15	EM and PEM physician	26	N/A	Weekly	Research in AR and VR
16	EM physician	19	Telehealth leadership and telemedicine fellowship director	Weekly	Research in AR and VR
17	EM physician	22	N/A	Never	N/A
18	PEM physician	15	N/A	Biannually	Simulation center leadership
19	EM physician	8	Tele-ICU^g^ director	Quarterly	N/A
20	PEM physician	0	N/A	Weekly	Research in VR
21	EM physician	8	N/A	Biannually	N/A

^a^AR: augmented reality.

^b^VR: virtual reality.

^c^EM: emergency medicine.

^d^N/A: not applicable.

^e^*Minimal* refers to the use of AR or VR technology in any context less than annually, but the respondent has personally used the technology at least once previously.

^f^PEM: pediatric emergency medicine.

^g^ICU: intensive care unit.

**Table 2:** Descriptions of themes and subtitles.

Themes and subthemes			Description
**AR^a^ in remote information gathering and sharing**			
	Improving the efficacy in observational tasks	Headsets with AR could provide perspectives and tools that improve visual tasks performed by providers and nonproviders compared with those provided by conventional telemedicine platforms.	
	Convenient access to data	AR would allow access to real-time information and references that expedite clinical decision-making.	
	Communication with experts	AR would facilitate information exchange with specialists and consultants.	
**AR in remote education**			
	Procedural coaching	Procedural guidance could be enhanced using AR tools and occur in contexts ranging from the operating room to beyond the hospital.	
	Nonverbal cues	AR technology could facilitate the teaching of skills related to cue recognition and empathy.	
	Connecting remote learners to local programs	AR technology could enhance learning across long distances between less experienced learners and specialized educators.	
**Barriers to the implementation of AR**			
	AR may increase existing disparities	Providers expressed concern that AR-enabled telemedicine may worsen known disparities in wealth, resources, literacy, and so on.	
	Providers need clinical value and support in adoption	Providers are hesitant to adopt new technologies without evidence of benefit and support at both the institutional and infrastructural level.	
	Providers anticipate consumer preferences to affect acceptability	Providers felt that public adoption and awareness will influence when they and their patients adopt AR.	

^a^AR: augmented reality.

### Theme 1—AR in Remote Information Gathering and Sharing

#### Overview

Emergency medicine providers collect and process information about many aspects of every patient they encounter. Participants perceive the addition of AR tools to potentially increase the efficacy of performing observational tasks, efficiency of accessing data and references, and ability of users to receive input from experts.

#### Subtheme 1.1—Improving the Efficacy of Observational Tasks

Compared with a traditional camera, providers expressed that a device that offered the point of view of a patient or caregiver in a different location would provide additional angles useful in a visual examination, especially for those who lack medical training:

As a telemedicine provider, the two-dimensional nature of interacting with patients on a screen is challenging. When we are at the bedside and want to be more mobile, I still need this cart that I got to wheel around or a phone that I have somebody hold up for me and turn, and that is primarily the limiting factor of why people don’t like telehealth right now...If you change that by just asking them to put on an augmented reality headset, and then I can have that shared point of view, there’s no holding a camera, there’s no wheeling anything into tight spaces...we can immediately get to the heart of the matter, and I can see what I need to see.

Participant 19

Some interviewees mentioned that features such as annotation or zooming in the field of view can assist in focusing a patient’s or caregiver’s attention and would be useful in collecting photographs and measurements. An interviewee described the following:

If you can point at an area, like “Look, I think I see something here. Can you, in this circled area, can you zoom in, get a picture and send it over to me...” Because sometimes, if the video is moving, you have a hard time seeing images. If they take a photo and send it over and from places of interest, I think that might be useful.

Participant 9

Interviewees also mentioned the uses of AR in observational tasks beyond those performed by providers, such as observation done by a patient unable to leave their room or by their relatives when in-person visits are not possible. A participant described how AR could enhance the visual experience of visiting family members, thus allowing more engagement in the patient care process despite remote circumstances:

You can actually bring in family, who are not there, to be part of the healthcare. So they can feel like they’re part of it. They do virtual rounds now over video, but you can do this in a much more involved fashion, so that the person doesn’t have to sit there the whole time, but the patient can feel like they’re there.

Participant 8

#### Subtheme 1.2—Convenient Access to Data

Respondents commented about AR’s potential to facilitate clinical decision-making by providing real-time, simultaneous access to past medical information and graphics while interacting with a patient, rather than accessing the electronic medical record (EMR) at a separate station:

A lot of medical decision making isn’t made on one data point alone. It’s made out of a conglomeration of data points, lab work, exam history, complaints, things like that. And so the AR part of it, where I’d be able to see all of those contexts on the same screen at the same time while seeing the patient, can be of utility in the telemedicine visit... [Participant 1] 

Interviewees also described the potential of AR to allow concurrent access to reference images or guidelines that they would normally access through the internet, such as specific physical examination findings, anatomy, or expected ranges for laboratory test values:

There’s the ability to, rather than me running to go and try to look at my computer or look at my phone, to say “okay, what are some images of that again?” or “is this what Bell’s palsy looks like? Well, let’s pull up a picture of what Bell’s palsy facial weakness looks like.” And it’s right there, right at the eyeball. Assistance for clinical decision-making and for education.

Participant 5

The seamless integration of AR with internet-connected tools that collect clinical data was also of interest. Examples of information that could be displayed in the AR-enhanced field of view include real-time vitals from monitors and results from electrocardiograms, sonography, and stethoscopes at the patient’s location. An interviewee stated the following:

We call them peripherals. Can I hear their heartbeat? Do I have a stethoscope? Can I listen to their lungs? These are features that would be great. So I could hear it myself rather than just going by secondary signs...If I could get the Apple Watch technology, to place that on and just see what’s going on for them, I think that’s where we’re at with this.

Participant 13

A few respondents commented about the role of artificial intelligence, in conjunction with AR, in identifying the appropriate data to display.

#### Subtheme 1.3—Communication With Experts

Respondents mentioned the use of AR in facilitating inpatient consultations and commented about the potential for patients to access hard-to-reach specialists as outpatients. Interactions with more experienced experts could yield advice that supplements the information used for medical decision-making. Remote specialty consultants could obtain footage of the patient and provide clinical input or instructions for a specific process such as resuscitation or a stroke code. Respondents also commented about the potential for interpreters and tool specialists (technicians specializing in ventilators, pacemakers, surgically implanted devices, etc) to provide support remotely through the use of AR:

Think smaller institutions that don’t have all the consults available to them. A lot of city hospitals don’t have ophthalmology hanging out in the middle of the night. That would be interesting, if you could do dilated eye exams...having people not come in the middle of the night, but still having the ability to get to the patient’s bedside and having a good point of view to do physical exams.

Participant 11

Respondents also described remote health delivery services that could integrate AR to allow remote experts to better support local staff, including emergency medical and disaster response services, teleparamedicine, telerehabilitation, and telewound programs. Few respondents mentioned uses in psychiatric care and less conventional settings including military, in-flight aircraft, and space.

### Theme 2—AR in Remote Education

#### Overview

In the context of remote learning, interviewees felt that the addition of AR could enrich education beyond the teaching of subject matter, such as procedural and nonverbal skills including cue recognition and empathy. Furthermore, the integration of AR tools could enable long-distance training programs, including international training.

#### Subtheme 2.1—Procedural Coaching

Respondents commented about the use of AR for providing active feedback, step-by-step guidance, and planning, with a participant describing how AR’s ability to display hand gestures provided additional feedback alongside existing communication modalities:

One of the opportunities that AR provides is that you can augment your verbal instructions with gestures, and those gestures are in the field of view of the local person...You have to think about how you add verbal, nonverbal and gestures together to enhance communication and jointly complete a task. They may be individually powerful but together, the most powerful tools.

Participant 16

Most of the interviewed participants (15/21, 71%) described the use of AR in teaching unfamiliar processes to the less experienced individuals, whether through interactive simulations and supervision of a trainee or allowing the trainee to shadow an experienced provider from a different location. Approximately half of the respondents (11/21, 52%) considered AR to particularly enhance simulations of high-risk case scenarios that are of low frequency. A respondent described how AR would allow instructors to remotely guide learners in real-time and gauge where the learner’s attention is focused without interrupting the simulation experience:

One of the things that AR can help with is teaching thought process. Let’s say we do a simulation, and I have the learners going through a case. The only way I can really get into their thought process is by stopping the simulation and incorporating a “talking about it after the fact” kind of thing. Or pausing the simulation and talking about it. With AR, I can see what they’re looking at, and I can perhaps annotate in real-time. I can introduce labs in real-time. I can gesture and point them to look somewhere else. I can coach through the encounter, which allows them to continue to work through the case, and I can see what they’re seeing.

Participant 1

Among the most cited contexts for AR in remote procedural learning are both general and specialized telesurgery and minor interventions including those nonspecific to emergency medicine, such as intravenous access. An interviewee stated the following:

You may have a PA and a surgeon remote. You may have a hospitalist and then an intensivist remote...That was the classic example of just telemedicine in general. So AR with telemedicine, you could screw that on. Interventionally then, you could do anything better essentially than what you’re already doing via telemedicine. You could walk someone through like ventilator settings or frankly, do actual true physical interventions. Like put a central line in, intubate someone, take the gallbladder out.

Participant 6

In addition, some respondents described use cases for AR in labor and delivery.

#### Subtheme 2.2—Nonverbal Cues

Respondents saw the capability of AR to help educators teach remote learners, both nonmedical and medical, about the important cues to recognize, especially with the ability of AR to create persisting labels and instructions in the environment. Rather than relying on verbal instruction, AR allows the learner to visually experience and later recognize a previously unfamiliar signal as a finding associated with a specific outcome or danger. For those without medical training, such as patients and caregivers, AR-assisted visualization of specific signs or symptoms may better inform them about the outcomes to expect and the required action:

The general categories are education, prevention, ongoing instructions and management. A lot of times when we’re discharging people from the emergency department, we’ll say to people, “Return if something worsens.” So I think even to illustrate signs and symptoms that should raise a flag or alarm them, that they need to get evaluated. [Participant 7] 

Nonphysicians could also use AR remotely to teach cue recognition, with a respondent describing how patients could learn to recognize fall hazards at home:

Maybe the patient puts on some special glasses, and then the occupational therapist can see everything that the patient sees, and then the patient is kind of just walking around their house...and the occupational therapist can be looking at all these different features of the environment that might be a fall risk, and then advising them on how to reorganize.

Participant 21

In the case of health care trainees, remote teaching goes beyond foundational knowledge such as anatomy, as some respondents commented about the use of AR to provide experience-focused learning through empathy. Through AR, trainees could identify unspoken signs or even experience the circumstances that affect a patient’s health. A provider explained the following:

For a third party to receive instruction, I think it would be nice to be able to see things from another person’s perspective...There’s this concept called embodiment that I think we really need in the health care setting in order to better understand your patients and the people around you.

Participant 20

#### Subtheme 2.3—Connecting Remote Learners to Local Programs

Interviewees saw the potential for AR in long-distance education programs and global health, with the most cited example being teleultrasound. Respondents also mentioned the increased capability and capacity of remote hospitals and long-term care facilities as a result of AR enhancing what advanced centers can share with less specialized facilities. A participant described how rural hospitals could expand their services to avoid transferring patients:

Rural areas do not have enough specialists. You can get full-on neurology—we talk about stroke all the time doing the full neuro exam, but they [rural areas] are doing it. You could probably even keep more patients than they already do. They keep a lot of patients within their hospitals already with telestroke programs and with this, can take it even further.

Participant 8

### Theme 3—Barriers to the Implementation of AR

#### Overview

The adoption of any technology requires overcoming challenges related to the users, infrastructure, and technology itself. The interviewed providers described barriers that apply to both AR and innovations in general. First, the addition of AR to telemedicine may potentially exacerbate existing disparities. Second, providers require both evidence of clinical value and support to adopt AR. Finally, providers view the acceptability of AR to also be influenced by consumer preferences.

#### Subtheme 3.1—AR May Increase Existing Disparities

Respondents described how the addition of AR to telemedicine would underscore the social determinants of health that already stratify patients in using general telemedicine, such as technology literacy, financial costs, equipment availability, and access to high-speed internet. Differences in technology literacy among providers may also lead to unequal care, as providers with more familiarity and access to AR devices may use them more effectively than others. An individual asked the following:

Do you want your 60-year-old attending that’s been practicing for 20 years doing this because they’re the most experienced provider? Or do you want your intern, who’s spent their life playing video games and using Oculus, doing this? Even though they don’t have the same clinical knowledge?

Participant 4

Concerns more specific to AR devices include those related to users with disabilities or susceptibility to motion sickness and the responsibility of cost, storage, and upkeep of AR devices. Given that AR may require unique hardware to be used, participants described how some populations that already struggle to access health care may also lack the means to fix and receive education about how to properly care for the device. Some commented about whether it would be necessary to develop dedicated facilities to overcome barriers regarding storage space, maintenance costs, and financial limitations of patients that prevent travel. However, the space needed to use AR may also be challenging to establish in rural areas that lack access to specialized health care or in densely populated urban areas where dedicated spaces would be costly to create.

#### Subtheme 3.2—Providers Need Clinical Value and Support in Adoption

Interviewees described that studies in the areas of efficacy, safety, patient satisfaction, and profit would influence providers’ acceptability of new technologies such as AR. Respondents described that efficiency also played a role, especially when considering the burden of wearing or being seen with an AR headset; difficulty in building rapport with patients; time consumed to set up tools; and availability of preexisting, less complex methodologies. An interviewee described the following:

Actually having a clinical benefit is not enough. Clinical benefit, and it either needs to make me more money or make my day easier and more efficient. You need one plus one, and ideally you need all three. Otherwise, adoption will be heavily limited. [Participant 5] 

Respondents also commented about the need for use infrastructure and institutional support, particularly with the use of AR tools occurring at an early stage of a provider’s training:

I think if you start this in med school, obviously you’re going to get a higher rate of acceptance that we can grow into a residency, and then attendingship, just like ultrasound. I grew up when ultrasound was still beginning, and now you can’t imagine being without it. I’m still lousy at it because I didn’t grow up with it in residency...

Participant 17

#### Subtheme 3.3—Providers Anticipate Consumer Preferences to Affect Acceptability

Interviewees commented that consumer adoption and awareness about AR would play a role in the acceptability of AR in health contexts, with some interviewees drawing comparisons with technologies adopted by consumers such as smartphones and VR devices:

I think we’ve shown that well-designed technology is engaging and can easily be adopted by large numbers of people. We all have mobile devices now—we didn’t have them 15 years ago and I think that the barriers are going to be how well the technology is developed to be intuitive and engaging. It needs to be at the point where it’s the way an Apple product is, right?

Participant 3

Interviewees anticipate mixed responses from providers and patients to AR in telemedicine, with one-third (7/21, 33%) of the respondents expressing a sense of inevitability for AR to appear in the future of remote health care, if given time:

For all technology adoption, there’s the early adopters and then on the other side, the laggards...They’ll be exceptions and it’ll get to the point where it’s so effective and makes you so efficient, and your job so much easier that you can’t not use it, and at that point the laggards will join and I just, personally, believe that point is somewhere within 10 to 20 years from now...When the pandemic hit, everybody was like, “you can use telehealth because it works and it’s great” and “we really need to use this,” and so they just snap their fingers and let everybody use it. So something like that will happen again when the people basically just demand it.

Participant 19

## Discussion

### Principal Findings

We conducted semistructured interviews with emergency medicine providers with various levels of experience in telemedicine and AR technology to understand the opportunities and concerns for the intersection of the 2 aspects. We identified several areas of interest in which AR can assist in conducting remote medical communications, including the process of information collection and dissemination in various clinical settings. Furthermore, we identified multiple considerations for the implementation of AR, including barriers and facilitating factors both common to health care innovations and unique to AR.

Although modern platforms used in telemedicine allow both audio and video communication, the video aspect does not always replicate the experience of human vision in usual in-person interaction. The ability of AR-enabled headsets and devices to provide a *see-as-I-see* perspective to remote users prompted the interest in using such technology to enhance the sharing of observed information. According to our interviews, this enhanced interaction facilitates evaluation and supervision, especially for step-by-step processes requiring active feedback, including procedures inside and beyond the operating room. Surgical specialties have explored this potential extensively, with telesurgery tools such as *Proximie* and *Virtual Interactive Presence and AR* allowing remote experts to see the operating field of surgeons and interact using real-time annotation and labeling [36-39]. The nonsurgical specialties have also seen an emergence of AR to share views for remote shadowing in inpatient wards [40,41] and wound evaluations [42,43]. For emergency medicine, studies have shown that AR assists both remote emergency procedures and assessments through the use of a shared view, with trials focused on speed and performance [29,44,45]. Results so far suggest that AR increased accuracy in triage [44] and performance scores in cricothyroidotomy compared with the unassisted procedures [29], with more studies needed to assess AR’s efficacy in other processes. Beyond the coaching of procedures, our interviews also suggest AR’s role in enabling nonphysicians to examine or observe individuals in otherwise inaccessible locations. Future studies involving the tasks performed by nurses, technicians, and patients would provide further insight into AR’s ability to enhance other health care services.

Access to useful views of the local user is not always sufficient, as swift clinical decision-making also requires the timely review of relevant past information and standard references. From our study, one of AR’s potential advantages is its real-time access to essential data that are conventionally obtained from a workstation or device elsewhere. Few studies have explored AR’s role in displaying visual aids to facilitate remote services related to emergency telemedicine [30,44], but the use of reference images and models with AR has appeared in other specialties, including telepathology [21], teleultrasound [46], and telesurgery [47]. These studies demonstrated how AR benefits information collection by enabling simultaneous, hands-free access to pertinent references without compromising the quality of the task performed. Our interviews also address the possibility of AR directly assisting in image and data collection, with some previous studies implementing AR tools to obtain photographs [48] and measurements, such as skin lesions [49] and child size [50]. However, the implementation and efficacy of such tools in a real-time, remote interaction require more studies. Despite AR’s potential to expedite and supplement the types of information gathered in a health care context, our interviews also emphasize the many barriers to AR’s incorporation into telemedicine systems that must be addressed: establishing long-distance communication and connecting to databases such as EMRs require a reliable internet connection that resource-poor areas lack, in addition to adequate equipment. Although updates to infrastructure and the introduction of 5G may address these barriers, our study also describes how future users will also require early training and user-centered design to navigate the simultaneous streams of data.

According to our study, AR is also perceived to enhance telecommunication with a remote expert capable of providing further insight or instruction crucial for health care delivery and education. Given the diversity of pathology encountered by emergency medicine providers, access to specialty opinion is paramount to providing accurate, quality care while creating the opportunity to expand the consulting provider’s capabilities. Some studies so far that have implemented AR technology for communicating with remote specialists involve telestroke [51], teleultrasound [52], and mass casualty event triage [44,45]. With sufficient fidelity and latency of the technology, the consultation in these settings was perceived to be feasible and reliable when comparing remote conditions with in-person conditions. Although the studies suggest that the AR-enabled interaction was noninferior in short-term outcomes, our results emphasize a need for further studies into the financial and long-term consequences faced by the remote users, particularly to investigate whether the variety of remote services and patient capacity are affected. In our study, providers defined clinical value to include not only financial and clinical benefit but also convenience. Given that emergency medicine physicians have been identified to use the most telemedicine to communicate with other health care professionals compared with other specialties [4], studies on the efficiency of AR in telemedicine is of interest, specifically in comparison with preexisting, less technologically advanced communication modalities.

Another potential use perceived by interviewees involved the educational opportunities between the more and less clinically experienced users, as they use AR to interact remotely—the literature includes numerous papers on AR in surgical telementorship and training [53,54] and few papers in contexts involving procedures outside the operating room [46,55]. Although the same quantitative measures are not consistently measured across these studies, favorable results in qualitative measurements such as ease of use and perceived efficacy have suggested that users are interested in using AR for remote learning. The role of AR in the education of trainees has also been previously investigated for anatomy [56,57], simulation [58,59], and social skills such as communication [32,60], with such applications increasingly appearing in the remote setting owing to interest in distance learning options [61,62]. Although our interviews reiterate the benefits of AR for medical trainees, they also suggest the use of AR in remotely teaching those without professions in health care. A recent study describes the use of AR in stroke education for patients [63], whereas a review of AR in overall patient education yields few studies, even though its results demonstrate positive ratings in patient satisfaction, interest, and comfort in AR as a learning tool [64]. Despite the potential for AR to facilitate teaching over large distances for a variety of populations, the literature so far and this study’s results suggest that the emergence of AR in telemedicine would likely appear in provider-to-provider contexts before provider-to-nonprovider contexts. Potential areas of AR research could involve the differences in feasibility and acceptability across different types of communicating dyads in health care.

### Future Directions

Regardless of the variety of uses that AR technology may offer for telemedicine, further investigation is needed to overcome the barriers that prevent its integration into general health care and education. Our study underscores the concerns regarding obstacles related to known social determinants of health (access to care, environment, finances, etc) and limitations of the current infrastructure and design of AR devices. For those introducing AR into health care, the research necessary to support its adoption extends beyond potential clinical and educational benefits. Evidence of patient satisfaction and financial gains is also perceived to be essential, with an example of the latter being the use of AR-enabled devices for telecommunication to conserve personal protective equipment [20,65,66]. Future, large studies can focus on which factors are most valued and whether factors change depending on the type of provider using AR. Furthermore, overcoming the costs of implementation, both material and nonmaterial such as time and training, is perceived to require action from not only the innovators and medical institutions introducing the technology but also the consumers using it. Consumers’ familiarity with AR is perceived to play an important role in its adoption—AR remains as a nascent technology, and its acceptability is limited by the lack of standardized, intuitive designs and concerns about technology literacy gap. Consumer-focused studies that examine readiness and explore perceived barriers to AR can inform future technological design. The discussion of AR in this study and related literature focuses on HMDs such as smart glasses, which are less ubiquitous compared with personal laptops, smartphones, and tablets. As such, the adoption of AR in telemedicine may first appear on these monitor-based devices rather than on HMDs. A review (conducted in 2014) of AR in health care education found that approximately half of the 25 included papers used mobile laptops [67], whereas another review (conducted in 2019) of AR in medicine found smartphone and tablet devices as the platform in 18 and 28 out of 338 publications, respectively [18]. Additional studies on how AR tools built for existing personal devices can enhance telemedicine may yield findings that are more representative of future implementations compared with studies introducing novel systems that are less widely available.

Another area of potential investigation involves the question of whether AR serves as an adjunct tool versus an independent, novel means for communication. Telemedicine providers often juggle multiple applications while engaging with the other user, such as the audio-video platform itself, EMR, and other internet-related databases the provider may use. Unifying these sources of data into a single interface has yet to be achieved, and the addition of a new *adjunct* such as AR would likely encounter resistance from a user population whose attention is already divided. This study emphasizes AR’s potential to improve efficiency in acquiring and exchanging knowledge, suggesting that an AR-enabled, portable communication device that integrates clinical decision-making tools into a single space would offer advantages over the current, stationary workstations and pose a unique direction for innovation. Past devices such as Google Glass had the potential to contribute to many in-person and remote medical services but had limitations such as limited video quality and battery life that prevented further adoption [68]. A device that overcomes these barriers without compromising ergonomics and the efficiency of its user may potentially reshape how health care is delivered, but many challenges unrelated to technology and access remain, such as navigating a slow-to-change medical infrastructure and ensuring patient privacy [69].

### Limitations

This study has several noteworthy limitations in its design. First, the interviews were conducted over the internet with brief video demonstrations of tools that interviewees would likely find unfamiliar. This form of elicitation, rather than an in-person session using the equipment, may have influenced their responses. Of note, despite only seeing 2 examples of AR to remotely interact with an at-home patient and caregiver, interviewees responded with a wide spectrum of settings and users when prompted. A second limitation relates to the population of study; participants were mostly practicing emergency medicine physicians recruited from urban, academic medical centers in the northeast region of the United States. Therefore, the perspectives of those interviewed in this study may not be generalizable to other regions; specialties; and different types of health care workers, such as nurses, technologists, and hospital administrators, to name a few. Several studies focusing on the caregiver, patient, and trainee perspectives of AR devices for specific telemedicine-related uses have shown positive acceptability [70-72], but few studies have explored the range of applications and obstacles for AR perceived by these nonphysician populations. Furthermore, more studies are needed to explore the perspectives of those who operate in settings unaffiliated with large hospitals, such as those in rural areas and providers in private practice. Third, the sample represented variable histories with telemedicine and AR or VR technology. Although responses between the extremes of experience levels share similarities, further exploration at the lower end of the spectrum (limited experience with telemedicine and AR or VR technology) may yield differing or even new perspectives.

### Conclusions

We conducted semistructured interviews with emergency medicine providers to identify areas of interest and possible challenges for AR in telemedicine. Overall, 2 common themes that emerged included uses for AR to improve remote information collection and distance education. AR enhances the collection of observational and medical data through its ability to share another individual’s perspective and access references and consultants, all of which facilitate active feedback and clinical decision-making. AR supplements distance learning by enhancing step-by-step procedural guidance and teaching of important cues, with educational opportunities available for interactions among providers and between providers and nonproviders. Participants recognized multiple barriers to implementation, some familiar to telemedicine and others specific to AR devices, and the need for institutional support and consumer familiarity to facilitate adoption. On the basis of these findings, we discuss future directions for the research and design of AR devices in the remote, real-time health care setting.

## Appendix

Multimedia Appendix 1 Interview questions and probes.

Multimedia Appendix 2 Video demonstration of a prototype using augmented reality features to guide a pedal edema assessment.

Multimedia Appendix 3 Interview codebook.

## Abbreviations

AR: augmented reality
EMR: electronic medical record
HMD: head-mounted device
VR: virtual reality

## Data Availability

Data sets generated during this study, such as deidentified transcripts, are available from the corresponding author upon reasonable request.
