# Supplementary material for: Perceptions About Augmented Reality in Remote Medical Care: Interview Study of Emergency Telemedicine Providers
Source: JMIR Form Res. 2023 Mar 28;7:e45211. doi: 10.2196/45211 (PMC10131657; doi:10.2196/45211)
Supplement: Multimedia Appendix 1 [file formative_v7i1e45211_app1.docx]

| **Topics** | **Stem Question** | **Probe** |
| --- | --- | --- |
| Medical Background | Introduce yourself and your roles in healthcare. | How long have you practiced medicine? |
|  | How long have you used (synchronous, audio-video) telemedicine in your practice? |  |
|  | How much of your practice is/was telemedicine? | What percent of your patient care is/was telemedicine? |
|  |  | What percent of your clinical and non-clinical work is/was related to telemedicine? |
|  | What do/did you use to practice telemedicine? | Number of platforms/software |
|  |  | Types of devices |
| AR/VR Experience | Describe AR and VR. |  |
|  | When was the first time you heard of AR and/or VR? | In what context? |
|  |  | Since then? |
|  | How often do you use AR and/or VR technology? | In what context(s)? |
|  | Have you encountered AR/VR technology in a healthcare setting? | In what context(s)? |
| Video Elicitation Questions | What are your initial thoughts? |  |
|  | What are your thoughts on this feature? | The provider saw the patient through the caregiver’s perspective. |
|  |  | The caregiver saw a virtual representation of the provider demonstrating actions or pointing to areas of interest. |
|  |  | The provider drew circles over landmarks on the patient for the caregiver to see. |
|  |  | There was an image of an annotated leg that the caregiver can use as reference. |
|  | What issues do you foresee with using the discussed features? |  |
|  | Are there other features that could enhance remote communication that you wish existed? |  |
| Perceptions of AR in Telemedicine | In what telemedicine contexts would AR be impactful? | Outpatient |
|  |  | Inpatient |
|  |  | Education |
|  |  | Interventional/Therapeutic |
|  | What barriers exist in using AR with telemedicine? | For providers? |
|  |  | For patients? |
|  |  | How can these barriers be overcome? |
|  | How do you think users will react to using AR with telemedicine? | Providers |
|  |  | Patients |
|  | Are there other factors that could affect the integration of AR with telemedicine? |  |
|  | Was there anything else you wanted to share? |  |
