# Supplementary material for: Perceptions About Augmented Reality in Remote Medical Care: Interview Study of Emergency Telemedicine Providers
Source: JMIR Form Res. 2023 Mar 28;7:e45211. doi: 10.2196/45211 (PMC10131657; doi:10.2196/45211)
Supplement: Multimedia Appendix 3 [file formative_v7i1e45211_app3.docx]

| **Subtheme** / Code | |
| --- | --- |
| **1.1 Improving the efficacy in observational tasks** | Headsets with AR offer more angles than fixed cameras  AR features are useful for the medically naive eye  AR features allow user to focus attention to area of concern  Potential for AR to take photos and recordings  Potential for AR to allow zooming in for measurements  Potential for patients and relatives to spectate remotely |
| **1.2 Convenient access to data** | Real-time access to health record  Potential integration of AI to display relevant data  Real-time access to reference guidelines or standards  Potential to access data from internet-connected devices and monitors |
| **1.3 Communication with experts** | Access to specialists as outpatient  Inpatient consultation of specialists  Obtaining input for a specialty-specific exam or process  Access to tool-specific experts  Access to interpreters  Emergency medical services can connect to remote experts  Teleparamedicine  Telerehabilitation  Telewound services  Remote therapy and psychiatric care  Access to healthcare in military, space and aircraft context |
| **2.1 Procedural coaching** | AR allows provision of active feedback  AR allows step-by-step guidance  AR allows procedural planning  Role in simulation education  AR enhances supervision of process  AR enhances shadowing of process  Role in training high-risk case scenarios  Coaching through minor procedures  Telementorship in surgery  Remote coaching in labor and delivery |
| **2.2 Nonverbal cues** | Annotation with AR creates persisting instruction  Role in teaching patients about condition and red flags  Potential for 3D models and anatomical learning  Potential for empathy learning |
| **2.3 Connecting remote learners to local programs** | Enables learning programs to reach remote areas  Teleultrasound training  Increases capability of remote institutions  Increases capability of long-term care programs |
| **3.1 AR may increase existing disparities** | Technology literacy  Financial costs  Difficulty in accessing equipment  Broadband requirements  Cannot be used by those with disabilities  Responsibility of maintaining equipment  Establishing and accessing dedicated facilities |
| **3.2 Providers need clinical value and support in adoption** | Adoption depends on proven benefits  Lack of research  Lack of evidence for financial benefit  Inconvenience of headset  Design not conducive to establishing rapport  Costs time to set up AR  Competes with existing methods  Lack of use infrastructure  Lack of institutional support  Need for early training in using AR tools |
| **3.3 Providers anticipate consumer preferences to affect acceptability** | AR lacks consumer adoption  Lack of awareness of AR in healthcare  AR devices need ease of use similar to existing consumer products  Anticipate mixed response from patients and providers  Emergence of AR in healthcare seems inevitable with time |
